# Low Radon Cleanroom for Underground Laboratories

**DOI:** 10.3389/fpubh.2020.589891

**Published:** 2021-02-02

**Authors:** Ivan Štekl, Jirí Hůlka, Fadahat Mamedov, Pavel Fojtík, Eva Čermáková, Karel Jílek, Miroslav Havelka, Rastislav Hodák, Miroslav Hýža

**Affiliations:** ^1^Institute of Experimental and Applied Physics, Czech Technical University in Prague, Prague, Czechia; ^2^SÚRO, Prague, Czechia

**Keywords:** radon, cleanroom, underground laboratory, nanotechnology, zero dose, equilibrium factor

## Abstract

Aim of a low radon cleanroom technology is to minimize at the same time radon, radon decay products concentration and aerosol concentration and to minimize deposition of radon decay products on the surfaces. The technology placed in a deep underground laboratory such as LSM Modane with suppressed muon flux and shielded against external gamma radiation and neutrons provides “Zero dose” space for basic research in radiobiology (validity of the LNT hypothesis for very low doses) and for the fabrication of nanoelectronic circuits to avoid undesirable “single event effects.” Two prototypes of a low radon cleanroom were built with the aim to achieve radon concentration lower than 100 mBq·m^3^ in an interior space where only radon-free air is delivered into the cleanroom technology from a radon trapping facility. The first prototype, built in the laboratory of SÚRO Prague, is equipped with a standard filter-ventilation system on the top of the cleanroom with improved leakproofness. In an experiment, radon concentration of some 50 mBq·m^−3^ was achieved with the filter-ventilation system switched out. However, it was not possible to seal the system of pipes and fans against negative-pressure air leakage into the cleanroom during a high volume ventilation with the rate of 3,500 m^3^·h^−1^. From that reason more sophisticated second prototype of the cleanroom designed in the LSM Modane uses the filter-ventilation system which is completely covered in a further improved leakproof sealed metal box placed on the top of the cleanroom. Preliminary experiments carried out in the SÚRO cleanroom with a high radon activity injection and intensive filter-ventilation (corresponding to room filtration rate every 13 s) showed extremely low radon decay products equilibrium factor of 0.002, the majority of activity being in the form of an “unattached fraction” (nanoparticles) of ^218^Po and a surface deposition rate of some 0.05 mBq·m^−2^·s^−1^ per Bq·m^−3^. Radon exhalation from persons may affect the radon concentration in a low radon interior space. Balance and time course of the radon exhalation from the human body is therefore discussed for persons that are about to enter the cleanroom.

## Introduction

Only recently, the need has been recognized in both nanotechnology (e.g., CCD image sensor, pixel detectors fabrication) and fundamental biological research (verification of LNT hypothesis for very low doses and without pollutants) to achieve at the same time very low radon and minimum aerosol concentrations in the air of laboratories, in so-called low radon cleanrooms (1). Radon and its decay products in the air or deposited on the surfaces emit alpha particles (with relatively high energy) which can cause undesirable “single event effects” in nanoelectronic circuits. In the fundamental research of biological cells response carried out in the radiation free environment (Zero dose in underground laboratories) radon and its decay products can adversely affect the experiment. Radon air concentrations are required to be as low as possible in such special cases. However, since levels of radon concentration in the outdoor atmosphere range normally from units to tens of Bq·m^−3^ and in buildings from tens to thousands Bq·m^−3^ the achievement of indoor radon air concentration in the laboratory at the level of some tens of mBq·m^−3^ requires advanced systems able to reduce radon concentration at least by a factor 1,000 in comparison to the outdoor environment. We present realization of the complex technology able to achieve at the same time low radon concentration levels of 10–100 mBq·m^−3^ for radon, about mBq·m^−3^ for radon decay products and the minimum aerosol concentrations in the air of the cleanroom as well as radon decay product ^218^Po deposition rate of some μBq·m^−2^·s^−1^. In addition, if the technology is placed in a deep underground laboratory such as LSM Modane with suppressed muon flux and the technology or at least a part of the inner space inside the cleanroom are shielded against external gamma radiation and neutrons, a complex suppression of the all above-mentioned components of external radiation can be achieved for Zero dose basic research.

## Prototype Setup

### Experimental Low Radon Cleanroom Technology—Prototype Development

Cleanroom is defined (ISO14644-1) as a room in which the concentration of airborne particles is controlled, and which is constructed and used in a manner to minimize the introduction, generation, and retention of particles inside the room and in which other relevant parameters, e.g., temperature, humidity, and pressure, are controlled as necessary. A clean room is generally based on several rooms in a row including e.g., room for person undressing, clean dressing room, air-shower where a strong air stream cleans the work clothes from adhering dust particles before entering to the clean room, and one clean room. The rooms are separately ventilated and filtrated. The person entering the cleanroom has to respect time required for air regeneration in each room. The doors are equipped with an alarm to indicate the current opening, the air shower door is equipped with a lock, preventing simultaneous opening. Thanks to all these procedures higher degree of purity is gradually achieved in adjoining rooms and the highest class of cleanliness is reached in the cleanroom intensively filtered by HEPA filters. The lowest aerosol concentration, however, is reached only in part of cleanroom just under air outlet from HEPA filtration system where the laminar flow is ensured. Standard commercial cleanrooms technologies don't deal with radon and therefore filtration system uses ambient air with outdoor background radon concentration.

**Figure 1:**
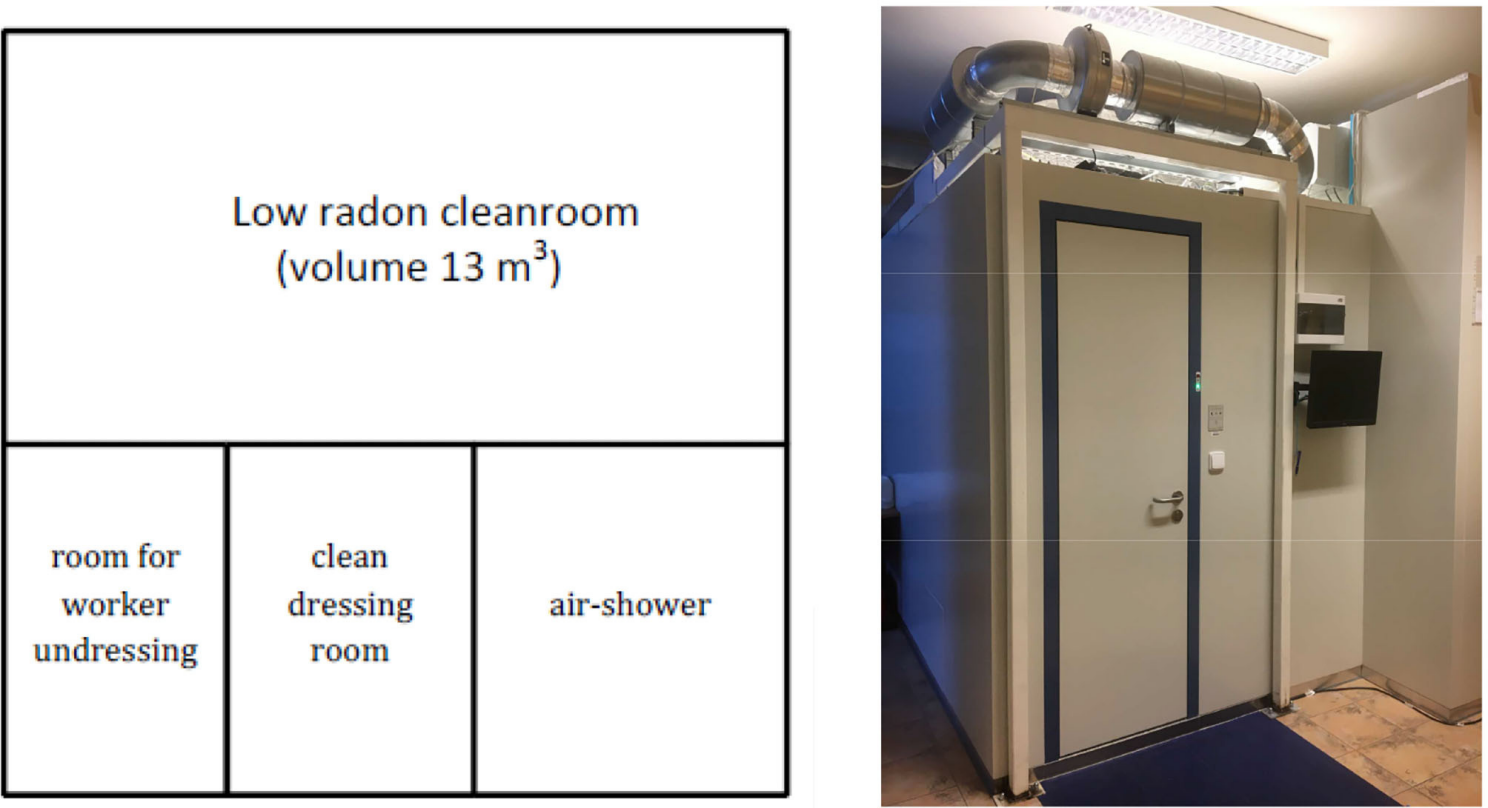
Radon Cleanroom at SÚRO laboratory, Prague.

The new technology developed to minimize radon and radon decay products in the cleanroom is based on following innovations: (a) the filter ventilation system is closed, it means the air from cleanroom returns back through HEPA filter into the cleanroom to avoid mixing with outdoor air, (b) the cleanroom uses air delivered from an external radon trapping facility, where adsorption of radon on charcoal takes place (2, 3), (c) radon exhalation from human body is taken into account. To reach minimal radon concentration in the cleanroom the “radon-free air” has to be delivered from radon trapping facility in a sufficient amount for two reasons: to ensure both sufficient air exchange rate for persons in the cleanroom and to ensure reasonable over-pressure to suppress potential radon entry from outer space. Commercial radon trapping facilities available today are able to deliver typically from 20 m^3^·h^−1^ up to 300 m^3^·h^−1^ “radon free air” with radon concentration in range 10–100 mBq·m^−3^·h^−1^. From that reason they are often used in underground laboratories for suppression of background radon concentration particularly for detectors in fundamental physical research, neutrino physics, astrophysics, such as NEMO-3 experiment (4). The low radon concentration in the air supplied from the radon trapping facility determines the achievable radon concentration in low radon cleanroom together with system leakproofness. An experimental prototype of the low radon cleanroom was built at SÚRO Prague laboratory. The prototype consists of four rooms (Figure 1), a room for person undressing (1), a clean dressing room (2), an air-shower (3), and a low radon cleanroom (4) intensively filtered by a closed filter-ventilation system (3,600 m^3^·h^−1^) with HEPA filters. The used experimental radon trapping facility is located out of the cleanroom and is able to provide to the cleanroom up to 20 m^3^·h^−1^ of radon-free compressed air by means of a cooper manifolds The air leaks out from the room through the air shower as well as in the ventilation system. Radon-free air is supplied directly into the cleanroom filtration system and allows to reach indoor overpressure at least 5 Pa. The air leaks out from the room through the air shower as well as in the ventilation system. Volume of the cleanroom is 13 m^3^ and the delivery of 20 m^3^ h^−1^ radon free air corresponds to the air exchange rate of ~1.5 h^−1^. Clean room meets the requirement of ISO 14644-1 (5) Class 5 (maximum concentration limits particles/m^3^ of air for particles equal to and larger than the considered sizes, it means 3 520 p/m^3^ < 0.5 μm, 832 p/m^3^ < 1 μm, 29 p/m^3^ < 5 μm). The standard room temperature is 20° C and can be changed, the standard relative humidity in the clean room is very low (<5% due to the dried air supplied from the anti-radon facility), but can be increased by humidifier with radon-free water.

**Figure 2:**
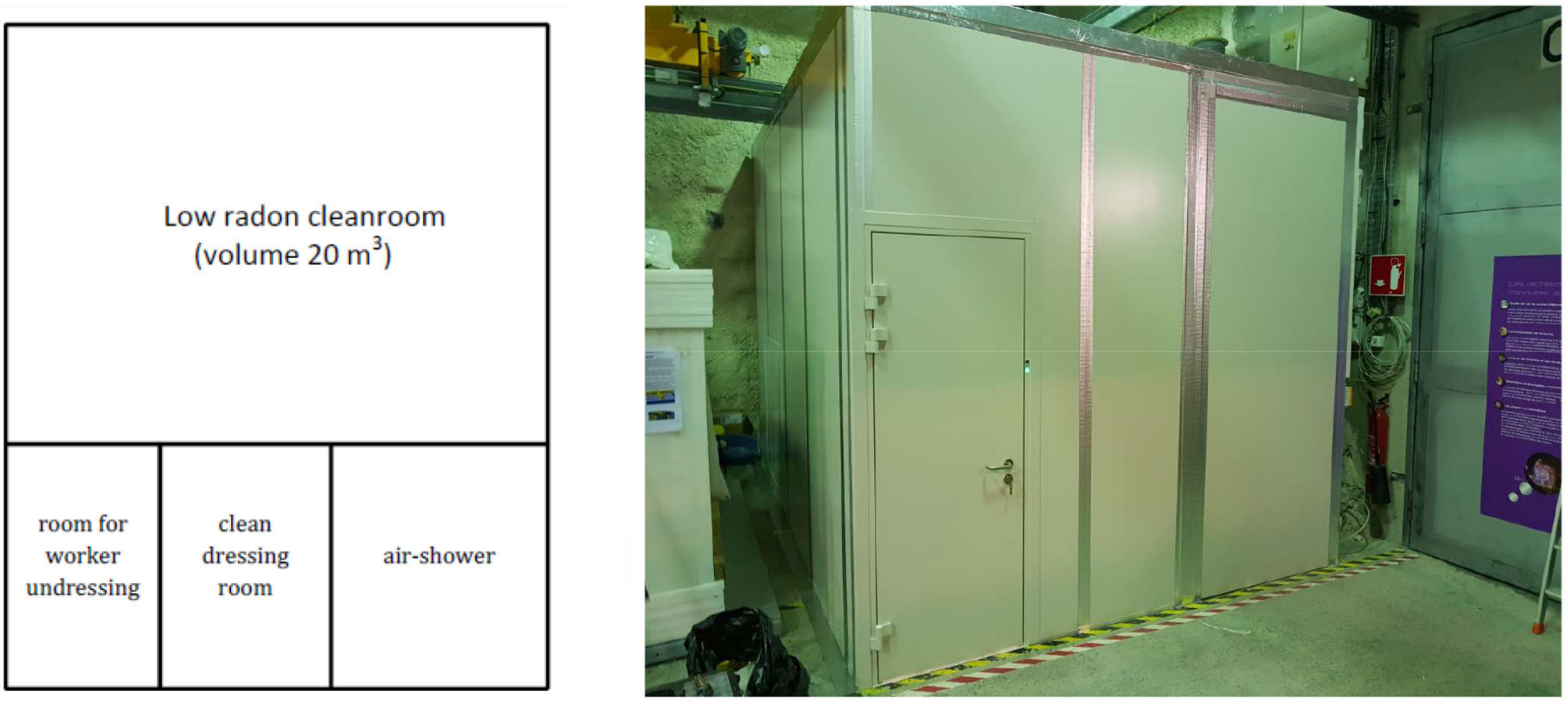
Radon Cleanroom at underground laboratory LSM Modane (2).

The goal of the prototype design was to reach indoor radon concentration in range 10–100 mBq·m^−3^. From that reason attention was paid to reduce so far as possible radon penetration from subsoil and ambient outdoor air and its emanation from building material. Hence, the whole room is built from metal (iron) and surface treated sandwich panels with thermal polystyrene insulation inside (total panel thickness of 62 mm). The panels are joined and sealed with sealant. To reduce penetration of ambient outdoor radon the panels are additionally sealed by a membrane with low diffusion coefficient (the TROPAC® foil). To reduce radon entry from subsoil, we have used metal plate directly placed and fixed onto the laboratory floor. It means that all walls, ceiling, and floor are made of a metal with high diffusion resistance for radon. In order to minimize radon/thoron exhalation from the surfaces into the room all material used for construction (iron sheet, thermal insulation, sealant) was selected and controlled in terms of the content of the relevant radionuclides, particularly for ^226^Ra and ^228^Th. The same control concerns to all objects located in the cleanroom (tables, instruments etc.). The limit for radionuclide concentration and radon emanation is determined by the demand for minimum radon concentration in the cleanroom with the available air exchange rate in mind. While air exchange rate is assumed to be above 1 h^−1^, the total radon exhalation from all the surfaces (walls, ceilings, floor, and objects inside) should be as low as possible in any case no more than some mBq·h^−1^ in order to not increase the radon concentration significantly. To reduce transport of contaminated air from the air wash to the low radon cleanroom a tight door between these rooms is used and in addition the room is operated at overpressure. In fact, the key factor influencing magnitude and time variation of radon entry from ambient outdoor air into the cleanroom is radon penetration through filter-ventilation system which is installed on the top of the whole cleanroom envelope. Leakages in filter-ventilation system and pipes were fixed step by step by mastic sealant and covered also by anti-radon TROPAC® foil. Experiments lasting several months were performed of experiments were performed in SÚRO laboratory to optimize system performance, study of the system tightness and pressure differences. Their results showed however, that it is not manageable to seal this complex engineering system of pipes and fans with high volume filter-ventilation rate creating a high under-pressure into the cleanroom. Only if the filter-ventilation system was switched off, it was possible to reach suppression of outdoor radon air entry into cleanroom by means of the created overpressure radon free air supplied from the external radon trapping facility. From that reason more sophisticated solution of next prototype of the cleanroom designed in the LSM Modane (Figure 2) uses the filter-ventilation system which is completely deployed in the tight sealed metal box placed at the top of cleanroom.

The last factor which can cause radon elevation in the cleanroom could be radon exhalation from persons entering the low radon cleanroom. This will be analyzed in discussion.

### Safety Elements and Equipment

Entire low radon cleanroom system is a closed system. The dried air from an anti-radon device guarantying an air exchange necessary for work (~1.5 h^−1^ in our prototype) is a single air supply. In addition to the standard safety elements, the system has been supplemented also with CO_2_ alarm sensor and camera for monitoring of the persons safety. Also humidifier with demineralized and radon-free water should be installed in case of long stay of persons inside.

## Results of Measurements

The first tests of the prototype of the low radon cleanroom built in SÚRO were focused on the

- tightness of the room
- radon gas measurement
- measurements of short-lived radon decay products and their unattached fraction
- measurements of deposition rate of radon decay products of and relevant surface contamination.

### Results of Ventilation Rate Measurement

The ventilation rate is the crucial parameter affecting behavior of all types of aerosol and gaseous contaminants (radioactive, chemical, biological) including radon and its short-lived decay product in rooms and buildings and moreover mediates also their convective transport from the outdoor environment to interiors of rooms and building. The ventilation rate can be found as a measure of the tightness of the room. The following simple and well-known mass balance model describes the effect of ventilation on the radon behavior in the room (6):

(1) $$\begin{array}{r} \frac{da_{i}\left(t\right)}{dt}=Qr\left(t\right)-\left(K\left(t\right)+\lambda \right).\left(a_{i}\left(t\right)-a_{0}\left(t\right)\right) \end{array}$$

*a*_*i*_*(t), a*_*o*_*(t)* is a time variation of indoor-outdoor radon gas activity concentration in (Bq·m^−3^)
*Qr (t)* is time variation of a sum of radon entry rates coming into the room in (Bq·h^−1^·m^−3^)
λ is defined decay constant for radon 0.00755 (h^−1^)
*K (t*) is time variation of ventilation (h^−1^)

To estimate ventilation of the room under a different operation conditions of the cleanroom (i.e., with the internal filtration system switched OFF and switched ON) we used so called tracer gas method based on both single injection and constant tracer gas entry into the room (7). In case of the use of the tracer gas method Equation (1) for radon gas can be modified as follows:

(2) $$\begin{array}{r} \frac{dc\left(t\right)}{dt}=G-\left(K\left(t\right)\right)c\left(t\right) \end{array}$$

*c(t)* is continually measured tracer gas concentration in the room in (kg·m^−3^)
*G* is constant, and well-known tracer gas entry in the room in (kg·h^−1^)
*K(t)* has the same meaning as in the previous Equation (1).

In case of the use of the single injection tracer gas method, the constant G is equal to zero in the Equation (2). For the constant gas method, time derivations $\frac{dc\left(t\right)}{dt}=0$ in Equation (2) can be substituted with a numerical differences. Ventilation was also calculated when the tracer reached the steady state concentration and member $\frac{dc\left(t\right)}{dt}=0$ in Equation (2).

As a tracer gas we used nitrous oxide(N_2_O) with defined and well-known entry rate into the room and we used a current sensor Polytron IR N_2_O (8) for continuous measurement of the tracer gas in the room (9). The results of multiple measurements indicated ventilation of the room ranged 0.015–0.02 h^−1^ for switched OFF filtration system and the same ventilation ranged 0.65–0.8 h^−1^ for switched ON filtration system with delivery of radon free air from the external radon trapping facility.

### Results of Radon Measurements

**Figure 3:**
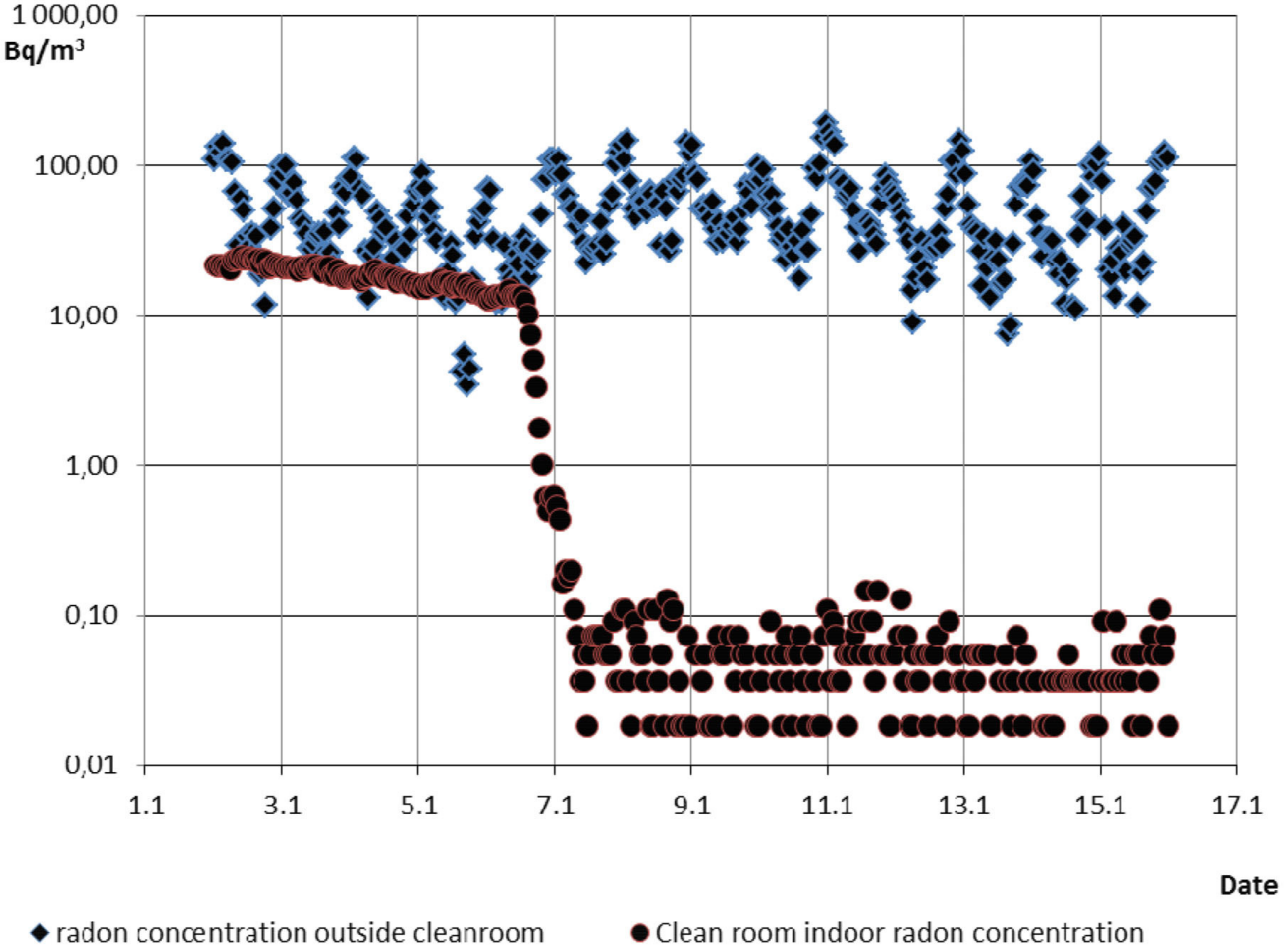
Comparison of radon concentration in laboratory and in cleanroom before and after switching on anti-radon facility.

During all radon tests radon gas activity concentration was simultaneously measured in the low radon room and outside the whole cleanroom in a SÚRO laboratory where is the whole cleanroom deployed. Radon concentration in this laboratory was measured continuously by TESLA Radon Probe TSR4 (10). Radon concentration shown in Figure 3 varied normally in the range of 10–200 Bq·m^−3^. Measurements of low radon concentration in the low radon cleanroom were carried out by a special high sensitivity (up to 10 mBq·m^−3^) continuous radon detector developed by IEAP CTU (11). After 2 years of experiments which included improvements in tightness and sealing of the system, proper overpressure in the low radon cleanroom and reasonable tightness between adjoining rooms (dressing room, air-shower, cleanroom) was achieved and radon concentration below 100 mBq·m^−3^ as is shown in (see Figure 3) was reached when the filter-ventilation system was switched off. The radon concentration mean value achieved in the low radon cleanroom was 52 mBq·m^−3^, while in the laboratory the mean value was 55 Bq·m^−3^ in the same period, it means radon reduction factor is more than one thousand. Time for exponential decrease of radon concentration to low level value after starting of radon-free air supply was ~6 h and is determined by air exchange rate 1.5 h^−1^.

### Results of Radon Decay Products Concentration and Equilibrium Factor

Investigation of the behavior of radon short-lived decay products was another goal of our tests. The key issue is concentration of short lived radon decay products ^218^Po (half-life 3.1 min), ^214^Pb (half-life 27 min), ^214^Bi (half-life 20 min) in the air and their deposition on surfaces and subsequent irradiation of sensitive objects by alpha particles. Generally, radon decay products are not in equilibrium with radon concentration in the room due to filtration and their subsequent deposition on the room surfaces. After their creation some decay products are attached to aerosol particles with diameter higher than ~10 nm (referred to as “attached” form). The rest is in the “unattached” fraction i.e., attached to clusters with a mobility diameter < ~10 nm (12). Radon equilibrium equivalent concentration (EEC) defined as activity concentration of radon in equilibrium with its short-lived progeny that would have the same potential alpha energy concentration as the existing non-equilibrium mixture is a simple quantity often used in human dosimetry for describing mix of short-lived radon decay products concentration (13). The ratio of EEC to the activity of parent radon in air is called equilibrium factor F and characterizes the disequilibrium between the mixture of the short-lived progeny and radon. Equilibrium factor F for indoor buildings is usually in the range (0.3–0.6) (14), it means approximately half of radon decay products are in indoor air.

The cleanroom is equipped with a filtration system of high filtration rate 3,600 m^3^·h^−1^. It corresponds with the mean time for filtration of the room (volume 13 m^3^) ~13 s which is much lower than the half-life of radon decay products. From that reason it could be expected that radon decay products will be very quickly removed from the air and equilibrium factor will be much lower than usual under normal conditions. In addition due to the use of a high efficiency HEPA filter in the room filtration unit a significant increase of a small clusters with only unattached ^218^Po can be expected. The results of such experiments for attached and unattached fractions of radon decay products are not known for such high filtration rate.

Currently, it is not easy to measure continuously short lived radon decay products in the air in case of low radon concentration ranging below 0.1 Bq·m^−3^. To estimate equilibrium factor and relationship between attached and unattached fractions of radon short-lived decay products all experiments had to be performed in an artificially high indoor radon concentration in the clean room during normal operation (huge filtration) so that radon and decay products are measurable with reasonable uncertainty. An artificial solid state ^226^Ra/^222^Rn flow-through source with ^222^Rn production rate certified in the Czech Metrological Institute was used. Initial high radon activity accumulated in the source was injected directly in the low radon cleanroom in operation followed by constant and defined radon entry rate. To achieve a steady state of radon and its short-lived decay products concentrations, the experiment lasted always a several days. During each exposure the built–in HEPA filtration system was operating and the air from radon-free facility was supplied to the room for maximum simulation of the real situation. While the steady state radon gas activity concentration varied in the order of kBq·m^−3^, the steady state activity concentration of measured unattached ^218^Po varied in the order of tens Bq·m^−3^ and attached ^218^Po varied in the order of units Bq·m^−3^. The amount of artificially added radon atoms in clean room is so negligible that cannot affect indoor condition of aerosol particles. All measurements were carried out with the continuous radon monitor AlphaGUARD and a continuous monitor FRITRA4 enabling simultaneous measurement of unattached and attached activity concentration each short-lived radon decay product from the open-faced filter and from the screen and the filter placed behind the screen. Measurement is based on the theory Cheng and Yeh (15) and Nazarof (16). Unattached activity concentration of each radon progeny was measured from the screen with mesh 300 and cut-off diameter d_50_ = 4 nm and the attached activity concentration of each radon progeny from a Millipore filter type AA, 0.8 μm placed behind the screen Jílek et al. (17) and Jílek et al. (14). Both instruments were calibrated in the Czech Accredited Calibrated Laboratory at Kamenná near Příbram (Czech Republic) as well as in the SÚRO Radon chamber (18). All the results indicated extremely low average equilibrium factor F ranging from 0.0015 to 0.0022. Nearly all radon decay products ^218^Po were in unattached fraction, average ratios of unattached ^218^Po and unattached + attached ^218^Po ranging from 95 to 98%. It could be deduced indirectly from this result that in case of a low radon cleanroom with reached indoor radon concentration of 50 mBq·m^−3^ the radon decay products concentration (EEC) of 1 mBq·m^−3^ will be approximately achieved with majority in the unattached fraction. This raises the question of what is the deposition on surfaces in such an unusual environment. Knowledge of this issue is important for research and technology fabrication in branches of nanoelectronic (to minimize “single event effect” by alpha particles), radiobiology (LNT hypothesis) etc.

### Radon Decay Products Surface Deposition: Measurement and Results

Two other preliminary experiments with artificially high indoor radon concentrations (89 and 1.4 kBq·m^−3^) in the cleanroom during operation of HEPA filtration system and ventilation by radon free air were performed to estimate deposition rate with reasonable uncertainty. An aluminum foil 1 m^2^ was placed in horizontal position in that part of the cleanroom with laminar flow of air. Foil was exposed 10 min to reach steady state activity on surface for deposited short-lived ^218^Po. The experiment was aimed at simulation of the manipulation with a metal surface, e.g., work with electronic circuits. The foils were measured by HPGe gamma spectrometry (deconvolution from ^214^Pb and ^214^Bi activity data set) at SÚRO laboratory. The deposition rate of ^218^Po was estimated 0.05–0.1 Bq·m^−2^·s^−1^ in case of indoor radon concentration 1.41 kBq·m^−3^ and 5–5.6 Bq·m^−2^·s^−1^ in case of indoor radon concentration 89 kBq·m^−3^. If related to unit indoor radon concentration of 1 Bq·m^−3^ estimated ^218^Po deposition rates are 0.036–0.07 mBq·m^−2^·s^−1^ per Bq·m^−3^ in first experiment and 0.056–0.06 mBq·m^−2^·s^−1^ per Bq·m^−3^ in second experiment, which is in good agreement. It can be again indirectly deduced that in case of indoor radon concentration 50 mBq·m^−3^ (achievable in contemporary low radon cleanrooms) the ^218^Po deposition rate will be ~3 μBq·m^−2^·s^−1^ and after the steady state is reached (as result of ^218^Po deposition and its decay) the surface deposition can be estimated as 700 μBq·m^−2^ (so below 1 mBq·m^−2^).

## Discussion

There should be mentioned another factor which may affect radon concentration in a low radon cleanroom. Persons exhale radon dissolved in their bodies by breathing mostly and become a source of an undesirable radon activity. The balance and time course of the exhalation from body is therefore of interest when persons are about to enter such premises. Before entering clean room the person must completely dress up into clean (radon free) clothes. Total radon activity in the human body depends on the radon concentration in the environment and time spent in it during last hours. Radon body content can range from units to some hundreds of Bq per body (19). Rapid radon exhalation from the body by breathing could cause increase of radon concentration in low radon cleanroom. The dynamics of the exhalation has been studied in the past by inhalation and ingestion experiments with ^222^Rn or with noble gases generally. Study of the ^222^Rn elimination from the human body subsequent to a relatively long-term inhalation was conducted (19). Recently, a biokinetic model for radon has been published (20, 21) based on theoretical approach and empirical data. The kinetics of radon exhalation decrease could be described by several exponentials, the first shows rapid decrease in exhalation by approximately a factor of 10 during 5 min and by a factor 100 during 2 h, etc. (19). The importance of the radon exhalation phenomena was also analyzed at SÚRO by experiments confirming Harley kinetic data. As example it can be estimated from these models that a person who spent some hours in concentration 1,000 Bq·m^−3^ exhales by breathing some 5 Bq·min^−1^ just after leaving this concentration what could influence significantly radon concentration in low radon cleanroom. Therefore, it is advisable to monitor the radon concentration in areas where persons reside before their entering to the cleanroom. If the concentration of radon is elevated, it is necessary to let the person in a low-radon environment for some time before entering the cleanroom. If the person in the cleanroom should not exhale more than some tens of mBq per hour (comparable to achievable radon concentration in low radon cleanroom today), for person who resides before at 100 Bq·m^−3^ it is necessary to spent ~2 h at low radon concentration only some Bq·m^−3^.There is also unavoidable radon exhalation from the body caused by natural ^226^Ra content in the body [below 1 Bq in skeleton, (22)]. In this case only a fraction of radon is able to emanate from the body and radon activity emanation attributable to ^226^Ra is approximately below mBq·h^−1^. The time needed to suppress the body radon exhalation up to levels that correspond with the inevitable radon exhalation due to natural ^226^Ra content in the body ranges a few days.

## Summary

A prototype of a complex technology able to achieve minimum aerosol concentrations in the air in terms of ISO standard (ISO 1999) and low radon concentration levels of 10–100 mBq·m^−3^ is available. From the measurements of attached and unattached fractions of radon decay products and from low equilibrium factor of F = 0.002 we can deduce indirectly that radon decay products equilibrium equivalent concentration (EEC) can be reached in the range some mBq·m^−3^, while majority of activity occurs in unattached (nanoparticle) fraction of ^218^Po. Preliminary results lead to an indirectly estimate that the deposition rate of ^218^Po is approximately some μBq·m^−2^·s^−1^ and deposition in steady state below 1 mBq·m^−2^ in the low radon cleanroom. A second improved prototype of a low radon cleanroom with improved tightness and delivery of 150 m^3^·s^−1^ radon free air was placed in the underground laboratory LSM Modane and is prepared for research and development. In case of further shielding against gamma and neutrons it is prepared to provide cleanroom or at lease space with nearly zero dose environment for fundamental experiments in biology, nanotechnologies etc. The zero dose environment does not mean a completely zero radiation background, but reduction of all radiation background components by several orders of magnitude (23). Specifically—reducing the radon concentration from typical values in the atmosphere or laboratories (in the range of 10–100 Bq.m^−3^) to tens of mBq. m^−3^ represents a reduction in the corresponding doses by a factor of 10^3^-10^4^. The muon flux (and dose) is reduced by a factor of 10^6^ The strategy to protect experiments in clean room against the neutron and gamma ray fluxes is standard: multilayer shields made of iron or clean Pb (20–30 cm), polythylene (16 cm) to thermalize fast neutrons followed by borated polyethylene (8 cm) to capture thermal neutrons. Such shields will surround the detectors or devices installed in clean room to reduce photon and neutron fluxes coming from the rocks of the laboratory. The gamma background can be reduced by a factor of 10^3^-10^4^, the neutron flux by a factor of 10^3^. It can be estimated that in LSM Modane the dose can be reduced by a factor of at least 10^3^, i e., significantly lower than 1 μGy per year.

The technology could be useful also for radiobiology e.g., validation of LNT hypothesis for very low doses, study of dosimetry models of radon risk. It is well-known from theory that the risk from radon exposure is much more affected by unattached radon decay particles rather than by attached particles and up to now there was no technology available for such experiments, as well as for the dark matter in CCDs experiment (24).

## Data Availability Statement

The raw data supporting the conclusions of this article will be made available by the authors, without undue reservation.

## Author Contributions

IŠ, JH, and EČ performed the substantial contributions to work conception and design low radon cleanroom. FM and RH were responsible for low radon concentration monitoring, detector development, and improvement and research of room tightness. IŠ, JH, PF, and KJ performed the substantial contributions to drafted and revised the manuscript critically for important intellectual content and provided the approval for publication of content. PF performed key contribution to radon exhalation from human body. FM, KJ, and MH were responsible for the acquisition, analysis, and interpretation of data. MH for spectrometry analysis of surface deposition of radon decay products. All authors contributed to the article and approved the submitted version.

## Conflict of Interest

The authors declare that the research was conducted in the absence of any commercial or financial relationships that could be construed as a potential conflict of interest.
